# Thymectomy in Juvenile Myasthenia Gravis Is Safe Regarding Long Term Immunological Effects

**DOI:** 10.3389/fneur.2021.596859

**Published:** 2021-02-25

**Authors:** Trine H. Popperud, Kiran A. Gul, Cathrine Brunborg, Richard W. Olaussen, Tore G. Abrahamsen, Liv T. Osnes, Emila Kerty

**Affiliations:** ^1^Department of Neurology, Oslo University Hospital, Oslo, Norway; ^2^Institute of Clinical Medicine, University of Oslo, Oslo, Norway; ^3^Pediatric Research Institute, Oslo University Hospital, Oslo, Norway; ^4^Oslo Centre for Biostatistics and Epidemiology, Research Support Services, Oslo University Hospital, Oslo, Norway; ^5^Department of Oncology, Oslo University Hospital, Oslo, Norway; ^6^Division of Pediatric and Adolescent Medicine, Centre for Rare Disorders, Oslo University Hospital, Oslo, Norway; ^7^Department of Immunology, Oslo University Hospital, Oslo, Norway

**Keywords:** juvenile myasthenia gravis, thymectomy, TREC, T cells, polyautoimmunity, immunosenescence

## Abstract

Thymectomy is an established treatment in adult MG and also recommended for the treatment of post-pubertal onset juvenile MG. Whether the youngest children should be thymectomized is still debated. Signs of premature aging of the immune system have been shown in studies on early perioperative thymectomy in children with congenital heart defect. In this retrospective cohort study the objective was to investigate the long-term effects of treatment related thymectomy on T cell subsets and T cell receptor rearrangement excision circles (TRECs) in peripheral blood of juvenile myasthenia gravis (MG) patients, as well as clinical occurrence of autoimmune disorders, malignancies and infectious diseases. Forty-seven patients with onset of myasthenia gravis before the age of 19 years were included; 32 (68.1%) had been thymectomized and 15 (31.8%) had not. They were studied at varying times after thymectomy (7–26 years). We found a significant lower number of naïve helper T cells (CD4+CD45RA+) with an increased proportion of memory helper T cells (CD4+CD45RO+), and a significant lower number of naïve cytotoxic T cells (CD8+CD27+CD28+) in the thymectomized patients. In addition they showed a significant reduction in the number of TRECs and proportion of recent thymic emigrants (RTE) compared to non-thymectomized patients. In none of them an increased frequency of malignancies or infections was found. Our findings indicate a premature aging of the immune system after thymectomy in juvenile MG, but associated clinical consequences could not be verified.

## Introduction

Juvenile myasthenia gravis (MG) is a rare autoimmune disorder giving fatigable muscle weakness due to immunological destructions at the endplate of the neuromuscular junction. In the majority of cases these attacks are mediated through autoantibodies against the acetylcholine receptors (AChR) at the endplate (1). Although B cells produce these antibodies; the destructive process is T cell dependent and also involves complement activation (2). It has long been established that the thymus plays an important role in the MG pathogenesis and the disorder is associated with thymic changes, both thymoma and thymus hyperplasia (3). Thymectomy has been used in the treatment of MG since 1941, but just recently the first randomized controlled trial on thymectomy in MG was conducted, and it showed benefit of thymectomy (4). The study did not disclose safety and efficacy findings in patients below the age of 18 years since these were excluded. Although there are no randomized controlled studies on thymectomy in juvenile MG, several prospective studies have shown positive effects (5, 6). In the latest international consensus guidelines for the management of MG, thymectomy is recommended for the treatment of postpubertal onset juvenile MG (7). It is still debated, however, whether the youngest children should be thymectomized. One of the questions raised is whether an early thymectomy has negative consequences for immune responses later in life. The weight of the thymus in proportion to the body is greatest just before birth. Atrophy and reduction of thymus activity start early in life, and its role after the initial T cell production is not clear (8). Studies on early perioperative thymectomy in children with congenital heart defects have shown signs of premature aging of the immune system, especially the T cell compartment (9–11). However, the clinical consequences are still not established.

Aging of the immune system is termed immunosenescence. T cell immunosenescence includes loss of thymus function, reduced number of recent thymic emigrants (RTE), proliferation of mature T cells and oligo clonal expansion of specific T cell subpopulations (12). Potential clinical implications are increased infection rates, reduced antibody response to vaccines and increased occurrence of autoimmunity and cancer.

We have in a previous retrospective study shown that thymectomy is efficacious in a Norwegian juvenile MG cohort including patients with prepubertal disease onset (13). The aim of the present study was to evaluate the long-term immunological consequences of thymectomy in juvenile MG especially focusing on association with age at thymectomy.

## Materials and Methods

### Patients and Blood Samples

In this population-based study, patient identification was conducted nationwide from January 2012 to April 2016 using multiple strategies: (i) through neurological and/or pediatric departments at the 15 main hospitals in Norway, (ii) through the national AChR antibody database at Haukeland University Hospital and (iii) through the national adult MG database at Oslo University Hospital. All patients had disease onset before the age of 19 years. MG diagnosis was based on clinical, serological, electrophysiological and pharmacological criteria described in detail in a previous publication (14). Retrospective clinical data were collected from medical records. Updated data on comorbidity and immunosuppressive treatment were collected through interviews at the time of blood sample collection for the T cell subset analysis. Blood sample collection was conducted at Oslo University Hospital from May 2015 to April 2016. Peripheral blood was collected by venipuncture from 32 thymectomized and 15 non-thymectomized juvenile MG patients. One sample was collected from each patient. The blood samples were analyzed for lymphocyte subsets and T cell receptor rearrangement excision circles (TRECs).

### Preparation and Quantification of T Cell Subsets

T cell subpopulations were analyzed by flow cytometry. Briefly EDTA-blood was incubated with optimally titrated antibodies for 15 min at room temperature, followed by erythrocyte lysis (BD FACS Lysing Solution, Becton Dickinson, San Jose, CA, USA). Data acquisition was performed on a Canto II flow cytometer (Becton Dickinson) and 100 000 cells was acquired when possible.

The following subpopulations were determined according to IPID (Immune phenotyping in Immunodeficiency), European Society of Immunodeficiencies.

T-cells were gated as CD3+ and further as naive CD4+ (CD4+ CD45RA+), recent thymic emigrants (CD4+ CD45RA+ CD31+), CD4+ memory (CD4+ CD45RO+), follicular like CD4+ (CD4+ CD45RO+ CCR5+), regulatory T-cells (CD4+ CD25+ CD127–), naive CD8+ (CD8+ CD27+ CD28+), CD8+ early effector memory (CD8+ CD27+ CD28–) and CD8+ late effector memory (CD8+ CD27–CD28–).

### TREC Analysis

DNA extraction was done using BLOOD DNA kit (Omega-Biotek, USA). The extracts were analyzed by PCR as previously described (15). To assure adequate DNA extraction betaactin was used as housekeeping gene. All qPCR assays had as required *R*^2^ values > 0.99 and similar slopes.

### Statistical Analysis

Demographic and clinical data are presented as either proportions or median values with interquartile range (IQR). Differences in categorical variables between thymectomized and non-thymectomized patients were tested by chi square test or Fisher's exact test as appropriate. Person's correlation coefficient (r) was used to analyze the association between two continuous variables. Linear regression analysis was performed to investigate the relationships between thymectomized patients and non-thymectomized patients and the total counts of T cell subsets, and to adjust for the possible confounding effect of chronological age. A significance level of 5% was used. All statistical analyses were performed using IBM SPSS Statistics for Windows, version 23 (Armonk, NY, USA: IBMCorp.).

### Ethics

The study was approved by the Norwegian Regional Committee for Medical and Health Research Ethics, South East Office. All patients, and also their parents when they were under 16 years old, gave written informed consent. Data were collected and registered in accordance with Norwegian guidelines.

## Results

### Clinical Data

Group characteristics and clinical data are presented in Table 1. The median follow up since thymectomy was 12 (IQR 7–26) years and median age at time of blood sample collection (chronological age) was 28 (IQR 25-41) years in the thymectomized (Tx) group and 24 (IQR 12-48) years in the non-thymectomized (non-Tx) group. At the time of the T-cell subset analysis, 12 patients received immunosuppressive treatment, 8 (25%) in the Tx and 4 (27%) in the non-Tx group. Autoimmune comorbidity was present in 11 (34%) of the Tx patients and in 6 (40%) of the non-Tx patients while malignancy only was reported in one patient, who was thymectomized. No patients reported increased frequency of infectious diseases.

**Table 1:** Clinical characteristics of the included JMG cases, thymectomized vs. non-thymectomized.

	**Thymectomized** ***n* = 32**	**Non-thymectomized** ***n* = 15**
Female gender, *n* (%)	25 (78%)	13 (87%)
Prepubertal onset^*^	10 (31%)	8 (53%)
AChR antibody positive MG, *n* (%)	29 (91%)	7 (47%)
Generalized MG, *n* (%)	30 (94%)	12 (80%)
Age at thymectomy in years, median (range)	17 (2–33)	n.a.
Thymus hyperplasia^**^, *n* (%)	20 (63%)	n.a.
Time from onset to thymectomy in months, median (IQR)	21 (9–31)	n.a.
Complete stable remission	16 (50%)	6 (40%)
Age at sample collection in years, median (IQR)	28 (25–41)	24 (12-48)
Time from thymectomy to sample collection in years, median (IQR)	12 (7–26)	n.a.
Immunosuppressives^***^, *n* (%)	8 (25%)	4 (27%)
Other autoimmune disorder, *n* (%)	11 (34%)	6 (40%)
Malignancies, *n*	1 (skin)	0

*IQR, interquartile range*.

* *Onset <12 years*.

** *Histologically verified at time of thymectomy*.

*** *Steroids, azathioprine, or mycophenolate mofetil at time of blood sample collection/t-cell subset analysis*.

*AChR, acetylcholine receptor; MG, myasthenia gravis; n.a, not applicable*.

### Reduced Number of Lymphocytes in Tx Patients

**Figure 1:**
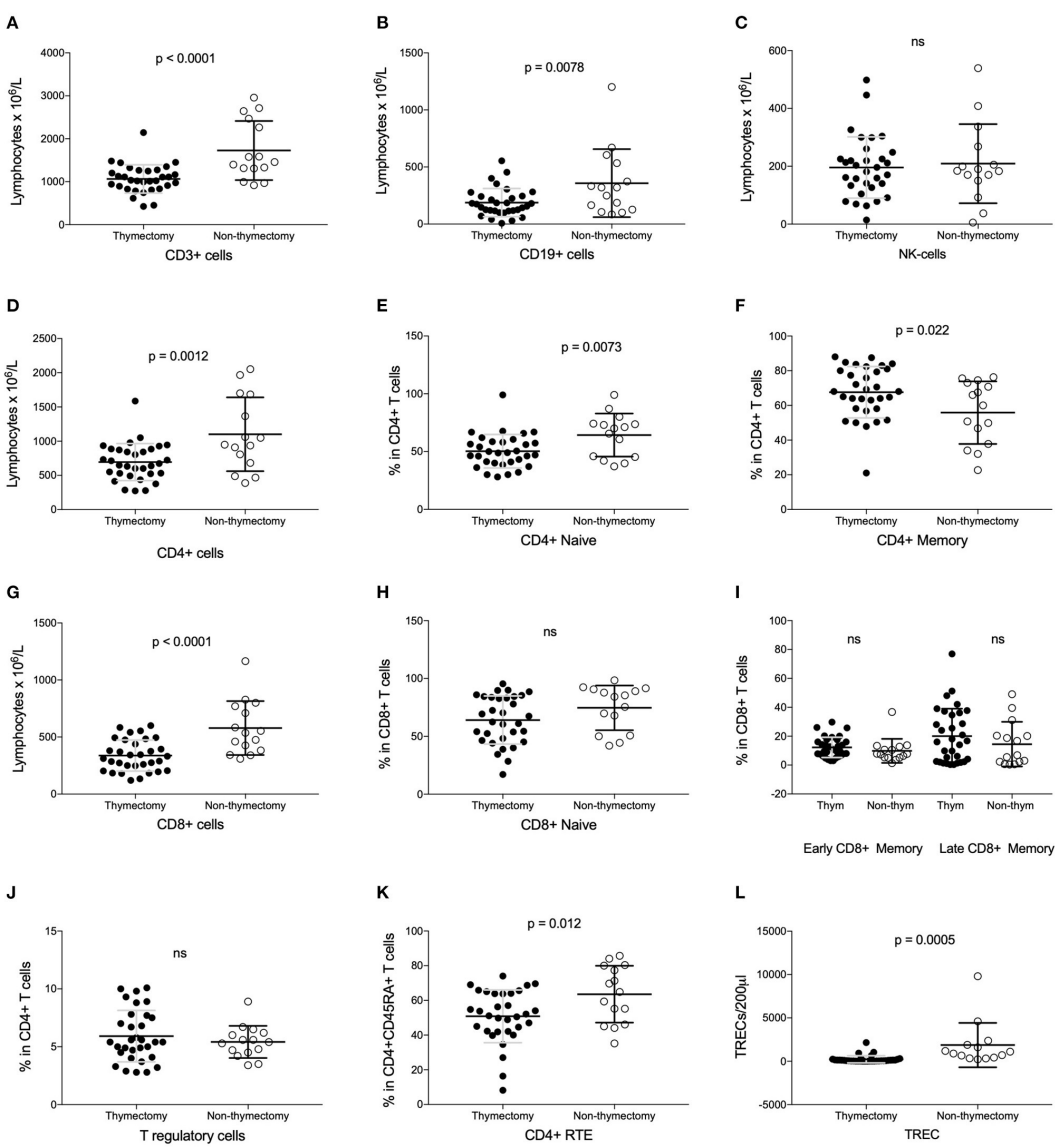
Analysis of lymphocyte subsets in thymectomized vs. non-thymectomized juvenile myasthenia gravis patients. **(A–D,G)** Number of CD3+, CD19+, NK, CD4+ and CD8+ cells. **(E,F)** Value of naïve and memory CD4+ cells as proportions. **(H,I)** Value of naïve, early/late memory CD8+ cells as proportions. **(J)** T regulatory cells as proportion of CD4+ cells. **(K)** Recent thymic emigrants (RTE) as proportion of naïve CD4+ cells. **(L)** TRECs per 200 μl. *ns*, not significant.

A reduced number of lymphocytes, both T cells (CD3+) (1066 vs. 1727 × 10^6^/L; *P* < 0.0001) and B cells (CD19+) (188 vs. 359 × 10^6^/L; *P* = 0.008), were found in the thymectomized patients compared to the non-thymectomized. Natural killer (NK) cells were unaffected. See Figures 1A–C.

### Reduction in Naïve CD4+ T Cell Numbers and Naïve CD8+ T Cell Numbers in Tx Patients

Looking more in detail at the T cell subsets, there were a decrease in the number of both CD4+ helper T cells and cytotoxic CD8+ T cells. See Figures 1D,G. The naïve helper T cell count (CD4+CD45RA+) was lower in the Tx group than in the non-Tx group, whereas the memory helper T cells (CD4+CD45RO+) were unaffected. However, the latter T cell subset count showed a proportional increase since the CD4+ helper T cell number was low. See Figures 1E,F. The number of naïve cytotoxic T cells (CD8+CD27+CD28+) was also lower in Tx cases, whereas the effector/memory cytotoxic T cells (early effector/memory: CD8+CD27+CD28-, and late effector /memory: CD8+CD27-CD28-) were unaffected by thymectomy, as were the regulatory T cells (CD4+CD25+CD127-). See Figures 1H–J. These associations were still present after adjusting for chronological age. We found no correlation of these T cell subsets with age at thymectomy (Table 2).

**Table 2:** Correlations of the T cell subsets of thymectomized patients with age at thymectomy.

	***r***	***p***
CD3	0.06	0.73
CD4+	0.05	0.77
Naive CD4+	0.01	0.96
Memory CD4+	0.07	0.69
RTE	0.08	0.64
CD8+	0.04	0.82
TRECs	0.01	0.96

*r, Person's correlation coefficient*.

*RTE, recent thymic emigrants; TREC, T cell receptor rearrangement excision circles*.

### Reduction in TREC Numbers and RTE in Tx Cases

Thymic activity was assessed by measuring TREC levels and recent thymic emigrants (RTE) by the surface marker CD4+ CD45RA+CD31+. TRECs (222 vs. 1868/200μl; *p* = 0.001) and the proportion of RTE (51 vs. 64% in CD4+CD45RA+ T cells; *p* = 0.012) were both lower in Tx patients (Figures 1K,L). The TREC and RTE values were negatively correlated with chronological age, *r* = −0.65 (*p* < 0.001) and *r* = −0.65 (*p* < 0.001), respectively. After adjusting for chronological age in patients, TRECs and RTE were still significantly lower in Tx group compared to the non-Tx group. There was no correlation of either TREC or RTE with age at thymectomy.

## Discussion

In this study we find that thymectomy in juvenile MG patients results in significant alterations in the peripheral T cell subsets, especially in the CD4+ subset with a decrease in naïve helper T cell with a relative increase in memory helper T cells, and a decrease in naïve cytotoxic T cells. These changes in the T cell compartment resemble findings characterizing the normal aging of the immune system (12), and they have been shown in other studies on children thymectomized while undergoing cardiac surgery (9–11). However, in an adult MG population neither lower total T cells, nor lower naïve T cells were found (16). Although the evidence of early T cell immunosenescence, there has been no clinical report of immunodeficiency following thymectomy in children during cardiac surgery, but the long-term clinical consequences are still incompletely revealed (9, 17).

Autoimmune comorbidity is a known feature in patients with myasthenia gravis, also in those with juvenile onset (13, 18, 19). Among our juvenile MG patients an autoimmune comorbidity was not more common in the Tx group than the non-Tx group, and in neither were malignancies nor infections frequently occurring (Table 1).

We could not show any effect on the T reg cells when comparing the Tx and non-Tx juvenile MG patients. Although a reduction of T reg cells have been shown after thymectomy in children undergoing cardiac surgery, several studies on adult MG patients have shown that T reg cells are not affected by thymectomy alone (20–22). Changes in T reg cells in MG patients are thought to be an effect of immunosuppressive treatment (21, 22).

TRECs and RTE were both lower in the Tx patients compared to the non-Tx patients. This illustrates the expected reduced thymic activity after thymectomy. This finding differs from an earlier study on thymectomy in adult MG patients where no difference in TREC numbers was found when Tx patients were compared with non-Tx patients. However, TRECs in both Tx and non-Tx patients were decreased compared to normal controls, suggesting an accelerated thymic atrophy in MG patients also independent of thymectomy (16).

Being a retrospective cohort study there are some limitations due to variability within the study population. The thymectomies were done at different ages, and the T cell subsets and TREC levels were measured at various time intervals after thymectomy. Earlier studies have shown a restoration of the T cell compartments with time after thymectomy, hypothesized to be due to thymic regeneration, and the regenerating capacity is speculated to be dependent upon age at thymectomy (23, 24). However, we found no correlation with age at thymectomy in our material. The AChR antibody positivity rate was higher in the Tx group as could be expected since thymectomy is more often advised in seropositive patients. Does this mean that the two subgroups are immunologically different? This question is beyond the scope of this study, but a longitudinal study on an adult MG population found no significant difference in levels of T cell subset after thymectomy in seronegative compared to seropositive MG patients (22). Approximately one fourth of the juvenile MG cases were on immunosuppressive medication. This was similar in both groups, and Sempowski et al. found that neither prednisolone nor immunosuppressive drugs affected TREC levels in their MG population (16).

## Conclusion

All though indications for a premature immunosenescence in the T cell compartment in thymectomized juvenile MG patients, we could not show any clinical consequences in our population at last follow up. The change in immunosenescence markers was not related to age at thymectomy. Thus, our study could not confirm any increased risk of thymectomy in prepubertal juvenile MG compared to postpubertal juvenile MG. The retrospective methodology, small sample size and variability within the study population however, are limitations of the study. Additional prospective studies including healthy controls are necessary to elucidate the effect of thymectomy in juvenile MG further.

## Data Availability Statement

The raw data supporting the conclusions of this article will be made available by the authors, without undue reservation.

## Ethics Statement

The studies involving human participants were reviewed and approved by REC South East. Written informed consent to participate in this study was provided by the participants' legal guardian/next of kin.

## Author Contributions

TP: study design, patient inclusion, analyses and interpretation, and manuscript writing. KG and LO: analyses, revision, and approval the manuscript. CB: analyses, revision, and approval the manuscript. RO, TA, and EK: study concept, data interpretation, revision, and approval the manuscript. All authors contributed to the article and approved the submitted version.

## Conflict of Interest

The authors declare that the research was conducted in the absence of any commercial or financial relationships that could be construed as a potential conflict of interest.
